# The Association between the Mediterranean Dietary Pattern and Cognitive Health: A Systematic Review

**DOI:** 10.3390/nu9070674

**Published:** 2017-06-28

**Authors:** Yasmine S. Aridi, Jacqueline L. Walker, Olivia R. L. Wright

**Affiliations:** School of Human Movement and Nutrition Sciences, The University of Queensland, Brisbane, QLD 4072, Australia; j.walker3@uq.edu.au (J.L.W.); o.wright@uq.edu.au (O.R.L.W.)

**Keywords:** dementia, Alzheimer’s disease, Mediterranean, diet, cognitive function, ageing, nutrition, cognition

## Abstract

The ageing population is accompanied by increased rates of cognitive decline and dementia. Not only does cognitive decline have a profound impact on an individual’s health and quality of life, but also on that of their caregivers. The Mediterranean diet (MD) has been known to aid in reducing the risk of cardiovascular diseases, cancer and diabetes. It has been recently linked to better cognitive function in the elderly population. The purpose of this review was to compile evidence based data that examined the effect of adherence to the MD on cognitive function and the risk of developing dementia or Alzheimer’s disease. This review followed PRISMA guidelines and was conducted using four databases and resulted in 31 articles of interest. Cross-sectional studies and cohort studies in the non-Mediterranean region showed mixed results. However, cohort studies in the Mediterranean region and randomized controlled trials showed more cohesive outcomes of the beneficial effect of the MD on cognitive function. Although more standardized and in-depth studies are needed to strengthen the existing body of evidence, results from this review indicate that the Mediterranean diet could play a major role in cognitive health and risk of Alzheimer’s disease and dementia.

## 1. Introduction

Dementia is the progressive decline of cognitive function, and this decline leads to a person’s inability to do routine daily activities. Most people living with dementia are within the elderly population; however, dementia is not a normal phenomenon when aging. Yearly, 7.7 million people are diagnosed with dementia, and, currently, there is no direct cure for it. Dementia rates are expected to triple to reach 135.5 million people living with dementia in 2050 worldwide [1].

Cognitive function is mainly related to knowledge and acquiring information. It is composed of various subgroups: attention, executive function, visuo-spatial skills and memory (short-term and long-term). All of these segments are essential components to cognition and knowledge. Attention compels the brain to focus on the important/relevant matters and reduce the effect of any interference. Executive function is how people use all the knowledge and skills they have in order to plan, organize and perform several tasks and solve problems [2]. Visuo-spatial skills are part of the short-term working memory, and are accountable for the storage and perception of visual and spatial stimuli. Long-term memory has no expiration date. Episodic memories are part of the long-term memory; it is where life episodes or events are used [2]. The Hippocampal part of the brain is the main part related to memories; it translates short-term memories into long-term ones. The hippocampus is responsible for episodic and spatial memory. Spatial memory is a form of object-location memory; it is spatial recognition like geographical knowledge and remembering an item’s location. In cases of dementia or cognitive decline, the hippocampal brain area is the first area to become impaired [3]. Given the numerous subgroups of the cognitive function, various cognitive tests have been developed to measure each subgroup. These tests could include questionnaires, blood tests, brain scans, personal history and a specific cognitive test or more than one test. Cognitive tests could range from a few minutes to more than two hours [4]. Many risk factors exist that can elevate the odds of developing dementia. The majority of these risk factors are reversible; however, others, such as, age and family history are irreversible. Reversible risk factors include: cardiovascular diseases (CVD), diabetes, depression, excessive alcohol intake, smoking, physical inactivity and poor dietary habits [5].

Some potential strategies to aid in slowing cognitive decline do exist; for example, several dietary strategies hold promise. Recently, data is emerging showing that the Mediterranean diet (MD) could aid in delaying the progression of cognitive decline; the Alzheimer’s society recommends the Mediterranean diet as an approach to improve memory and cognitive function [6].

The Mediterranean diet is the traditional dietary pattern followed by people residing on the shores of the Mediterranean Sea. Countries at the European shore include, Greece, France, Spain, Italy, as well as countries on the Southern shore such as, Egypt, Libya and Algeria. There is no unified consensus on what the Mediterranean diet is, for the minor details differ from one country to another depending on that specific culture; however, the key concepts are the same across all Mediterranean countries. Carbohydrates and starches, mostly unrefined, such as, bread, pasta, cereal, bulgur, potatoes and grains are at the base of the pyramid. Cheeses, yoghurt, fruits and vegetables are consumed in abundance on daily basis among people in the Mediterranean region. Given that this dietary pattern is low in animal protein; towards the top of the pyramid one will find chicken, fish and eggs; these foods are not consumed more than a few times a week. Traditionally, red meats are rarely consumed in this population; no more than a few times a month. The Mediterranean diet is not considered a diet that is low in fats; fat content ranges from 28–40% of the total dietary intake. However, it is low in saturated fatty acids and trans fatty acids due to its low content of animal meats and processed foods. Olive oil, oleic acid, a beneficial monounsaturated fatty acid is the major contributor to this fat content which makes it a healthy dietary pattern regardless of its high fat content [7].

Various MD scoring criteria are established to assess adherence to the MD. The two most used scores are: Trichopoulou’s 0–9 score [8], and Panagiotakos’s 0–55 score [9]. Trichopoulou et al. in 1995 [8], established a scoring criteria that assessed adherence to the traditional Greek Mediterranean diet. The MD is characterized by a high intake of favorable foods such as fruits and nuts, vegetables, cereals (including bread and potatoes), legumes, and a low consumption of unfavorable foods such as meats/meat products, poultry and dairy products. In 2002, fish intake was also added to the list of favorable foods. To calculate the MD score, the median intake of each food group is calculated for males and females separately. Each food group is given either a score of 0 or 1. In total the Trichopoulou Mediterranean dietary score ranges from 0 (lowest adherence to the MD) to 9 (highest adherence to the MD) [8]. Panagiotakos’s 0–55 Mediterranean dietary score was established in 2006; this score was calculated from a self-administered food frequency questionnaire. Similar to Trichopoulou’s score, this score examines intake of various food products determine the final scoring; however, a total of 11 food groups are included in this criteria. Favorable food groups include: unrefined cereals, vegetables, fruits, legumes, potatoes, fish and olive oil; where as, unfavorable food groups include: meats/meat products, poultry and full fat dairy products. Each food group is given a score between 0–5 depending on the recommended intake in the Mediterranean dietary food pyramid. The total score of Panagiotakos’s MD score ranges from 0 (lowest adherence to the MD) to 55 (highest adherence to the MD) [9].

Several mechanisms have been proposed as to how the MD exerts a beneficial effect against the risk of cognitive decline. Firstly, the MD is rich in anti-oxidants, and this content has been shown to have a protective effect against cognitive decline. Some of the disease that have been linked to oxidative stress are cancer, atherosclerosis, CVDs and dementia. Due to its high content of fruits, vegetables and olive oil, the MD is abundant in anti-oxidants [8]. The ratio of reduced to oxidized glutathione (GSH/GSSG) is a common measure of oxidative stress. Dai et al. (2008) have shown that a 1-unit increase in MD adherence score has been shown to increase GSH/GSSG by 6.8% 95% CI (0.8–13.2), and people with the highest MD adherence scores had a 31% 95% CI (2.1–69.7) increased ratio [10]. Another study has shown that MD was inversely associated with two markers of lipid peroxidation, F2-isoprostane (8-iso-PGF2a), and 9-hydroxyoctadecadieneoic acid (9-HODE), (*p* = 0.01 and <0.001 respectively). MD was also associated with higher plasma ascorbic acid levels (*p* = 0.04) [11]. Increased levels of oxidative stress and lipid peroxidation have been shown to increase the risk of developing cognitive decline and AD [11,12,13]. Reactive oxygen species (ROS) lead to damaged DNA and eventually cell death, consequently leading to an increased rate of ageing and cognitive decline [14].

Secondly, the Mediterranean diet includes a high intake of fish and olive oil, both rich in omega 3 fatty acids that exert an anti-inflammatory effect [15]. The high fiber content in fruits and vegetables could also exert an anti-inflammatory response [16]. Lastly, red wine impacts a similar effect by decreasing levels of C-reactive protein (CRP) and interleukin (IL) [17]. Studies have shown that the Mediterranean dietary pattern has been associated with improved anti-inflammatory response. A randomized trial by Esposito et al. (2004), showed that participants following the MD had significantly lower serum levels of CRP and IL-6 (*p* = 0.01 and 0.04 respectively) [18]. Another study has shown that for a 1-unit increase in MD adherence score, CRP and IL-6 levels decrease by 3.1%, 95% CI (0.5–5.7) and 1.9%, 95% CI (0.5–3.4) respectively [17]. Long-term inflammation might damage the blood brain barrier, thus increasing the amount of pro-inflammatory reagents inside the brain, leading to cognitive decline and AD [19,20]. Results from a cohort study that included 1929 participants revealed that participants with coronary heart disease (CHD) had a higher risk of developing mild cognitive impairment, OR = 1.93, 95% CI (1.22–3.06) [21]. In another study, participants with heart failure had an increased risk of dementia and AD, HR = 1.84, 95% CI (1.35–2.51) and 1.80, 95% CI (1.25–2.61) respectively [22].

Lastly, cardiovascular diseases are a known risk factor for dementia; for example, atrial fibrillation leads to strokes. Strokes may cause neurological losses which in turn affect neurological function, one of which is cognitive function. Moreover, atrial fibrillation on its own can lead to decreased hippocampal volume, and affect both short and long term memory [23]. Similarly, heart failure leads to decreased brain volume and causes damage in brain cells due to hypoperfusion in the brain area [24]. Cardiovascular diseases and dementia have common risk factors such as hypertension, arterial stiffness, diabetes, obesity and smoking [25]. The MD has been shown to have a beneficial affect on CVD mainly by decreasing the risk of developing risk factors associated with CVD [26]. A meta-analysis of six trials showed that the MD was significantly associated with body weight −2.2 kg, 95% CI (−3.9, −0.6), serum cholesterol −7.5 mg/dL, 95% CI (−10.3, −4.4) and CRP −1 mg/dL (−1.5, −0.5); all of which are risk factors of CVD [27]. Another study showed that after 10 years of adherence to the MD, participants with the highest MD scores had a 26% lower risk (95% CI (0.61–0.90)) of developing myocardial infarction, and a 22% lower risk (95% CI (0.65–0.93)) of developing a stroke [28]. Grosso et al., in their meta-analysis of 11 studies, showed that participants with the highest MD adherence score had a significantly lower risk of developing CVD and CHD, RR = 0.76, 95% CI (0.68–0.83) and 0.72, 95% CI (0.60–0.86) [26].

The Mediterranean diet is one of the healthiest diets, and it has been linked to decreased risks of various chronic diseases such as, CVD, obesity, hypertension and diabetes [29]. In addition, most recently, MD has been linked to decreased risk of cognitive decline and dementia [30,31]. Given the worldwide increasing rates of dementia and the beneficial effect of the MD, the aim of this review was to compile literature based evidence, and to examine the effects of the Mediterranean dietary pattern on cognitive function and dementia. A secondary aim of this study was to analyze possible reasons for the discrepancies of results in the literature.

## 2. Materials and Methods

### 2.1. Literature Search

The electronic search of English articles was conducted in January 2017, and included articles published since the start of the literature until January of the year 2017. The following search engines were searched: Pubmed/MEDLINE, Sciencedirect, Scopus and EBSCOhost (databases: MEDLINE, Australia/New Zealand Reference Centre, CINAHL, Hospitality & Tourism Complete, Teacher Reference Center). The search key words were: (hippocampal volume OR hippocampus OR spatial memory OR cognition OR cognitive function OR dementia) AND (Mediterranean diet OR Mediterranean food). The following filters were used for Pubmed (language- English and species-human), Sciencedirect (field- title abstract and keyword), Scopus (field- title abstract and keyword), EBSCOhost (language- English, species-human, and full text/abstract available). Query translation in the search engine: ((hippocampal [All Fields] AND volume [All Fields]) OR (“hippocampus” [MeSH Terms] OR “hippocampus” [All Fields]) OR (“spatial memory” [MeSH Terms] OR (“spatial” [All Fields] AND “memory” [All Fields]) OR “spatial memory” [All Fields]) OR (“cognition” [MeSH Terms] OR “cognition” [All Fields]) OR (“cognition” [MeSH Terms] OR “cognition” [All Fields] OR (“cognitive” [All Fields] AND “function” [All Fields]) OR “cognitive function” [All Fields]) OR (“dementia” [MeSH Terms] OR “dementia” [All Fields])) AND ((“diet, Mediterranean” [MeSH Terms] OR (“diet” [All Fields] AND “Mediterranean” [All Fields]) OR “Mediterranean diet” [All Fields] OR (“Mediterranean” [All Fields] AND “diet” [All Fields])) OR (Mediterranean [All Fields] AND (“food” [MeSH Terms] OR “food” [All Fields]))). MeSH Terms for hippocampus: CA1 Region, Hippocampal, CA2 Region, Hippocampal, CA3 Region, Hippocampal, Dentate Gyrus, Mossy Fibers, Hippocampal, Fornix, Brain. MeSH Terms for cognition: Awareness, Cognitive Dissonance, Cognitive Reserve, Comprehension, Consciousness, Imagination, Dreams, Fantasy, Intuition, Metacognition. MeSH Terms for dementia: AIDS Dementia Complex, Alzheimer Disease, Aphasia, Primary Progressive, Primary Progressive Nonfluent Aphasia, Creutzfeldt-Jakob Syndrome, Dementia, Vascular, CADASIL, Dementia, Multi-Infarct, Diffuse Neurofibrillary Tangles with Calcification, Frontotemporal Lobar, Degeneration, Frontotemporal Dementia, Primary Progressive Nonfluent Aphasia, Huntington Disease, Kluver-Bucy Syndrome, Lewy Body Disease. The inclusion criteria included: English full text articles, articles with neutral to positive scoring on the quality assessment criteria, articles that assessed dietary intake and cognitive function using standardized and validated tools. The exclusion criteria included any non-English articles, abstract that did not have full-texts available, articles that included non-human participants, articles that did not focus on MD and cognition, and articles that scored a negative on the quality assessment criteria.

### 2.2. Data Extraction

Study information was assessed using the title, keywords, abstract, and when information was insufficient, full texts were also used. Titles and abstracts of all articles were skimmed for potential inclusion. Full text articles were downloaded for all potentially inclusive articles and revised for the inclusion criteria. Summaries of all potentially inclusive articles were then tabulated on a Microsoft Word doc. (Title/Authors/Journal; Quality; Objective/Hypothesis; Experimental Design; Outcome measure(s); Results; and Comments).

### 2.3. Quality Assessment

Articles were assessed using the Academy of Nutrition and Dietetics Evidence Analysis Library Quality Criteria Checklist for Primary Research. This checklist is divided into two sections assessing both relevance as well as validity of the study. The relevance section is composed of four “yes/no” questions, the study qualifies to the validity section only if all answers to section one are “Yes”. The validity section contains 10 questions, with various sub-questions in each one. Validity is based on several aspects of the study; such as, research question, participant selection, comparability in-between study groups, withdrawals, blinding method, validity and reliability of outcomes measured, statistical analysis method, conformity of conclusion to results and any reported conflict of interest. If the article scores a “No” on more than six question, it is labeled with a “−”; whereas, if most of the questions (including 4 particular questions) score a “Yes”, it is labeled with a “+”, otherwise, it is considered neutral and labeled with a “0” [32].

## 3. Results

### 3.1. Study Selection

**Figure 1:**
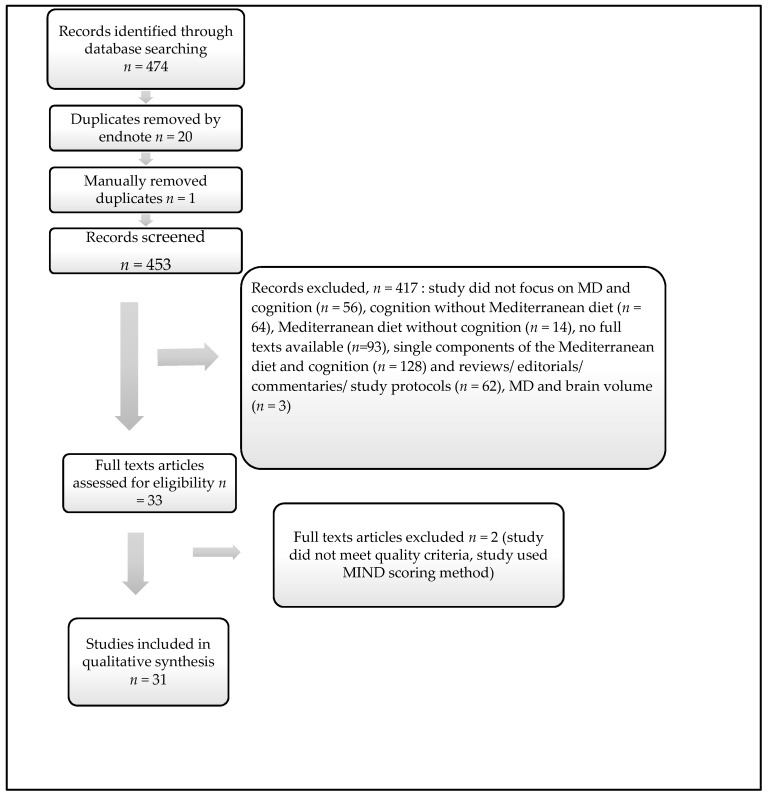
PRISMA preferred Reporting Items for Systematic Reviews [33].

This review followed the PRISMA guidelines for systematic reviews and meta-analyses [33]. The total results obtained at the time of the search were *n* = 193 for Pubmed/ MEDLINE, *n* = 41 for Sciencedirect, *n* = 57 for EBSCOhost and *n* = 183 for Scopus. All *n* = 474 references were exported directly to endnote, of which 20 were duplicates and removed automatically (Figure 1). Another manual search was conducted to removed duplicates not detected by the automatic search, *n* = 1. Afterwards 453 titles and abstracts were reviewed to assess relevance and eligibility. After reading through titles and abstracts, 417 references were removed for the following reasons: random articles (*n* = 56), cognition without Mediterranean diet (*n* = 64), Mediterranean diet without cognition (*n* = 14), no full texts available (*n* = 93), single components of the Mediterranean diet and cognition (*n* = 128), reviews/editorials/commentaries/study protocols (*n* = 62) and MD and brain volume (*n* = 3). Thirty-three full text articles were assessed for eligible, one of which did not meet the quality assessment criteria and the other study used the Mediterranean-DASH Intervention for Neurodegenerative Delay MIND scoring. Finally, 31 articles were included in this systematic review (Table 1). Detailed quality assessments for each of the articles included in the review are found on Supplementary Material 1. We were interested in comparing the association between the MD and cognition across Mediterranean versus non-Mediterranean region, and examine the efficacy of recommending the MD for people from other parts of the world. As a result, we decided to stratify the articles into Mediterranean versus non-Mediterranean study participants.

### 3.2. MD and Cognitive Function

#### 3.2.1. Cross Sectional Studies

Six cross-sectional studies examined the effect of adhering to the traditional Mediterranean diet on overall cognitive function. Three studies showed significant association [37,38,39]; however, the results of the other three studies did not detect any association between MD and memory [34,35,36].

(1) Studies that Detected Significance

The first study by Ye et al. [37] examined the association between adherence to the MD and cognitive function as part of the Boston Puerto Rican Health Study. The Mini-Mental State Examination (MMSE) assessed five areas of cognitive function: orientation, registration, attention and calculation, recall, and language. In all age groups, higher adherence to the MD was associated with higher MMSE scores, (*p* = 0.012) (Table 2) [37]. The second study was done by Zbeida et al. [39] and included participants from 2 cohort studies, the National Health and Nutrition Examination Survey (NHANES) and the Israeli National Health and Nutrition Survey (MABAT-ZAHAV). In both cohorts, the MD was associated with significantly better scores in the Wechsler adult intelligence scale. In the NHANES and MABAT ZAHAV studies, participants in the highest tertile of the MD score respectively had mean intelligence scores of 46.46 ± 18.12 and 31.27 ± 3.25, compared to means of 39.69 ± 18.52 and 30.41 ± 4 among participants within the lowest MD score tertile [39]. The only study that used Panagiotakos’s 00–55 MD score included 557 Greek participants aged 65+ years. MMSE scores were significantly positively associated with higher MD scores in men, and inversely associated in women, (*p* = 0.02 and 0.04 respectively) [38].

(2) Studies that Did Not Detect Significance

In a study that included Australian participants, cognitive function was assessed using a Cognitive Failures Questionnaire (CFQ) and a Memory Functioning Questionnaire (MFQ) that measure perception, memory, recalls and motor function. Adherence to the MD was not shown to be associated with the overall reported cognitive function [35]. A study in China included men and women, as part of a cohort study examining the risk factors for osteoporosis in Hong Kong. Community Screening Instrument for Dementia (CSI-D) test was used to measure memory, language function, visual context/visual-spatial cognitive function. Among both sexes, MD scores were not associated with cognitive function [34]. Lastly, a study that was part of the Lothian Birth Cohort 1936 Study (LBC1936) also used the 0–9 MD score and the MMSE, National Adult Reading Test (NART) and Wechsler Test of Adult Reading (WTAR) cognitive tests. The MD was associated with better verbal ability in both the NART and WTAR, *p* = 0.024 and 0.001 respectively. However, after adjusting for all possible covariates the MD was not shown to be associated with memory status, *p* = 0.870 [36].

#### 3.2.2. Randomized Controlled Trials

The two trials included 856 participants, with high cardiovascular disease risks and showed significant results. The first trial was part of the Prevención con Dieta Mediterránea (PREDIMED) study and followed up participants for 6.5 years. Participants followed either a MD with Extra Virgin Olive Oil (EVOO) (1 L/week), MD with nuts (30 g/day) or a control low fat diet. In both MMSE and Clock Drawing Test (CDT) (a measure of spatial dysfunction), participants in the MD with EVOO had significantly better global cognitive function compared to controls in the low fat groups (*p* = 0.005 and 0.001 respectively). Participants in the MD with nuts group, compared to controls, also had significantly better scores, (CI 0.11–1.03, *p* = 0.015 for MMSE, and CI 0.003–0.67, *p* = 0.048 for CDT) [40]. The second randomized parallel-group clinical trial took place in Barcelona between 2003–2009, and participants were randomly assigned to one of the following nutritional interventions: MD supplemented with either olive oil (1 L/week) or nuts (30 g/day), or a low fat control diet. Cognitive function was assessed using MMSE, Rey Auditory Verbal Learning Test (RAVLT) (short-term auditory-verbal memory), Animals Semantic Fluency (sematic memory), Digit Span subtest (short-term verbal memory), Verbal Paired Associates (episodic memory) and Color Trail Test (attention). Nonetheless, only the RAVLT and Color Trail Test showed significant results where participants in the MD supplemented with olive oil, compared to controls, had better scores, (*p* = 0.049 and 0.04 respectively) [41].

#### 3.2.3. Cohort Studies

(1) Studies in the Mediterranean Region

The first cohort study to link MD with cognitive decline took place in France in 2009 as part of the Three-City (3C) study. Cognitive function was assed using: MMSE, Isaacs Set Test (IST) (verbal fluency), Benton Visual Retention Test (BVRT), and Free and Cued Selective Reminding Test (FCSRT) (attention and acquisition); however, only the MMSE showed significant results over the 5 year follow up period. For every 1-point increase in the MD score, participants had fewer errors on the MMSE test (*p* = 0.04) [50]. The second study in France was part of the Supplementation with Vitamins and Mineral Antioxidants (SU.VI.MAX) study. Cognitive function was assessed by the the RI- 48 (Rappel indice’ (cued recall)-48 items) to assess episodic memory. While lower MDS scores were associated with lower backward digit span performance (*p* = 0.03), lower MSDPS scores were associated with lower phonemic fluency performance (*p* = 0.048) [51].

A more recent set of cohort studies were published in 2015; two studies used a Food Frequency Questionnaire (FFQ) [52,56] to assess dietary intake and calculate Trichopoulou’s 0–9 MD score. As part of the Greek European Prospective Investigation into Cancer and Nutrition (EPIC) prospective cohort study, participants enrolled between 1996–1999, were part of the final study. MMSE tests were done twice during the 6.6 years of follow up to measure cognitive function. Compared with participants with the lowest tertile of the MD score, those within the second and third tertile had respectively a 25% and 54% significantly lower odds ratio of mild cognitive decline and a 28% and 66% lower odds ratio of substantial cognitive decline [56]. In another study, the Nutritional aspect of the Spanish prospective cohort (SUN project) started in 2008, and 823 volunteers, aged 55+ years, participated in the study. Two Telephone Interview of Cognitive Status (TICS-m) were conducted to assess cognitive function. Participants in the bottom two tertiles of the MD adherence scores had higher cognitive decline than participants with the highest scores [52].

Only one study in the Mediterranean region did not detect a significant association between MD and cognitive health. This study included both males and females as part of the EPIC study in Greece. After 6 to 13 years of follow up, participants with the highest rates of adherence to the MD had no significant difference in MMSE scores as compared to those within the lowest adherence levels [48].

(2) Studies in the non- Mediterranean Region

The first study in a non-Mediterranean country took place in the USA, Tangney et al. and participants were from the Chicago Health and Aging Project (CHAP). Cognitive function was assessed by East Boston tests of immediate and delayed recall, MMSE and the Symbol Digit Modalities Test (attention, visual scanning, tracking and motor speed). Higher MD scores were significantly associated with slower rates of cognitive decline. Results remained significant even after people with the lowest baseline cognitive scores or heart diseases were excluded [54]. In another US cohort study, this time as part of the Reasons for Geographic and Racial Differences in Stroke (REGARDS) Study, results showed that higher adherence to the MD among non-diabetic participants was associated with 19% lower risk of incident cognitive impairment, OR 0.81; 95% CI 0.70–0.94; *p* = 0.0066 [58]. Another similar study cohort study; however, for a longer follow up period as part of the Cache County Memory Study (CCMS) showed similar results. Participants with the highest adherence to the MD, scored higher on the Modified MMSE (3MS) test as compared to those with the lowest adherence (*p*-trend = 0.0022) [57]. In a more recent study by Tangney et al., and as part of the Memory and Aging Project (MAP) cohort study that started in Chicago in 1997 results showed that for every 1-unit increase in MD score, the rate of cognitive decline was slower by 0.002 standardized units (SEE = 0.001, *p* = 0.01). However, only the upper tertile of the MD score was associated with slower decline rates in episodic, semantic, working memory and global cognitive change domains [55]. The final study in the USA was part of the Health, Aging, and Body Composition (Health ABC) prospective study. After 8 years of follow up, significant results were observed only among black participants; participants with higher MD scores had slower rate of cognitive decline (95% CI: 0.05–0.39 *p* = 0.01) [53].

Another cohort study showed significant results and also used TICS-m cognitive test. This study took place in China as part of the China Health and Nutrition Survey and included 1650 adults aged 55+ years. After following an adapted MD for 5. 3 years, the rate of cognitive decline decreased; moreover, participants with the highest adherence scores had a 0.28 decreased rate of cognitive decline as compared to those with the lowest adherence scores, 95% CI (0.02–0.54) [49].

The first study that did not detect significance took place in Canberra, Australia, and as part of the Personality & Total Health (PATH) Through Life study, and included both men and women. Participants administered the Clinical Dementia Rating scale (memory, orientation, judgment and problem solving) to assess cognitive function. However, MD did not have any protective effect against cognitive decline [42]. In another Australian study, results from the Australian Imaging, Biomarkers and Lifestyle study (AIBL) showed similar results. This study used the AusiMedi score, a score similar to Trichopoulou’s score but uses cohort sex specific median instead of the traditional sex specific median. MD was only associated with AD development among the APOE ε4 allele carrier but not among the general participants [47].

Four cohort studies in the USA; all of which only included female participants, did not show any significant association between adherence to the MD and cognitive decline. The first study was part of the Women’s Antioxidant Cardiovascular Study (WACS) and included 2504 female health professionals. During the 5.4 years of follow up, no significant results were observed between participants with different levels of adherence to the Mediterranean diet and their rate of cognitive decline [46]. Samieri’s study included 16,058 female nurses that underwent various cognitive tests: TICS (verbal memory, orientation/mental tracking, language/reasoning, and attention/working memory), immediate and delayed recalls of the East Boston Memory test (EBMT) (verbal memory), category fluency and digit span-backward. Results showed no significant association between adherence to the MD and cognitive decline over time. However, adherence to the MD was linearly significantly associated with better overall cognitive function (*p* = 0.004, 0.002, <0.001 for TICS, global cognition and verbal memory respectively) [44]. The second study also by Samieri et al. included 6174 women health professionals, recruited as part of the Women’s Health Study in the USA. Similarly, the results did not show any significant association [45]. The final study was by Haring et al., as part of the Women’s Health Initiative Memory Study (WHIMS), examined the effect of 9 years of following a MD on cognitive decline. No significant differences were observed across participants in MD adherence quintiles and cognitive decline over time [43].

### 3.3. MD and the Risk of Developing Alzheimer’s Disease

#### 3.3.1. Studies That Detected Significance

Five studies assessed the effect of adherence to the MD on the risk of developing Alzheimer’s disease; four of which showed significant results. The first study was published by Scarmeas et al. in 2009, and participants in this study were part of the Washington/Hamilton Heights Inwood Columbia Aging Project (WHICAP I–II). Participants in the third tertile of adherence to the MD had a 51% lower risk of developing Alzheimer’s disease, HR = 0.49 (0.29, 0.85) (*p* = 0.01) [60]. In a more recent cohort study by Morris et al., part of the Rush Memory and Aging Project (MAP) in Chicago, people with the highest adherence to the MD, highest tertile for MD score, had a 54% lower risk of developing Alzheimer’s disease, compared to those in the lowest tertile. This association remained significant even after adjusting for CVD, HR = 0.49 95% CI (0.29, 0.85) [63]. Similar to by Scarmeas et al., Gu et al. examined a subgroup of the WHICAP II study. Participants in the greatest adherence to the MD had marginally better cognitive results β = 0.013 (*p* = 0.05). After 4 years of following a MD, longitudinal analysis showed that participants within the highest tertile of adherence to the MD had a 34% less risk of developing AD [64]. Another cross-sectional study took place in Australia was part of the Australian Imaging, Biomarkers and Lifestyle Study of Ageing (AIBL) study and included participants that were either healthy, had mild cognitive impairment (MCI) or AD. Participants that had AD and MCI had lower MD score than cognitively healthy participants (*p* < 0.001 and <0.05 respectively). Every 1-unit increase in the MD score was associated with 13–19% and 19–26% decrease in odds in being in the MCI and AD category respectively. Out of the 4 cognitive tests used, only the MMSE test showed significant results; in which participants with the highest MD scores achieved best on the MMSE, *p* = 0.014 [59]. Lastly, results, from a study that included 192 individuals residing in New York, showed a protective effect of MD against mortality risk in the study population. Moreover, AD patients with higher MD adherence scores, compared to those with the lowest scores had a 74% lower risk of mortality, 95% CI = 0.10–0.69 [62].

#### 3.3.2. Studies That Did Not Detect Significance

The only study that did not detect a significant association between MD and AD development was a Swedish cohort study as part of the Uppsala longitudinal study of adult men. This study used a modified MD score, this score is similar to Trichopoulou’s 0–9 MD score but does not include nuts. After 12 years of follow up, the MD was not associated with AD prevention, HR = 1.00 95% CI (0.75, 1.33) [61].

## 4. Discussion

This evidence-based systematic review examined the efficacy of the Mediterranean diet against cognitive decline and dementia. Results from published articles show mixed results, especially among cross-sectional studies. Cohort studies show a discrepancy in the usefulness of adherence to the MD in studies taking place in the Mediterranean versus non- Mediterranean regions. However, cohort studies published in the Mediterranean region show encouraging results. These results are further reinforced by two Spanish randomized controlled trials that also showed promising results.

### 4.1. Variations in Dietary Scores and Cognitive Tests

All cross-sectional studies used Trichopoulou’s MD score except for one study that used Panagiotakos’s MD score. The two scoring criteria have been used intensively in the literature, and they have been found to be reliable and valid tools to assess adherence to the Mediterranean diet [8,17,65,66]. The usage of either of the two MD scores did not seem to affect the association between adherence to the MD and over-all cognitive function. However, given that different cognitive tests measure different domains of cognition; the choice of the cognitive test used might have affected this association. The three cross-sectional studies that showed a significant association used the following cognitive tests: MMSE and Wechsler adult intelligence scale. The MMSE is the most commonly used cognitive screening tool in research articles [67,68], and, compared to other brief tests, it is the best tool to detect dementia [69]. The MMSE is a measure of orientation, attention, calculation, recall, and language; whereas, the Wechsler adult intelligence scale is a measure of IQ. The three studies that did not detect significance used the CSI-D, CFQ/MFQ as well as MMSE. CSI-D measures: memory, executive function, language function, visual context/ visual-spatial; CFQ/MFQ measure memory, absent-mindedness, or slips of action, attentiveness. The CFQ/MFQ tests used by Crichton et al. (2013) and the CSI-D test used by Chan et al. (2013) were self-reported by participants in the study, this fact might have also affected the significance of the association between MD and cognition [34,35]. Another possible reason for not detecting significance in Crichton’s study could be that their study participants had the lowest mean age among all the cross-sectional studies. The MD might have a stronger effect on cognitive health later in life rather than earlier on. Additionally, all studies that used MMSE test reported significant results, except for one study by Corley et al. (2013) [36]. This particular study was the only cross-sectional study that accounted for childhood IQ and added it into their covariate analysis. People with better childhood IQ may be more health aware and tend to follow a healthier dietary lifestyle. The later shows childhood cognitive function and IQ could mediate and affect the link between MD and later life cognition.

### 4.2. Variations in Age and Follow up Duration

All cohort studies included participants with a mean age of at least 52 years; therefore, age did not seem to be a factor that affected the significance of the association between MD and cognitive function over time. Most cohort studies used Trichopoulou’s MD score. Studies that detected significance ranged in follow up periods between 4–13 years; similarly, studies that did not detect any significance had a ranged in follow up periods between 3–13 years (Table 3). However, the longest duration study linking MD to the risk of developing AD didn’t show any significance. In that particular study, researchers did not assess intake of nuts. Nuts are a substantial contributor to the benefits of the MD, and have been associated with lower risks of cognitive decline [40,41]. Both cohort studies that took place in Australia had the briefest follow up periods and did not show significant results [42,47].

### 4.3. Variations in Study Region and Gender

Out of the five cohort studies in the Mediterranean region, only one did not show a significant association. This study was published by Psaltopoulou et al. (2008) and a possible reason for not having significant results could be that they did not collect baseline data for their participants, all other studies had baseline data available [48]. In contrast, studies in the non-Mediterranean region showed mixed results; out of the 12 cohorts included in this review, six did not show significant results. It is important to note that four out of the six studies in the non-Mediterranean region that did not detect significance included only female participants (Table 2). This shows that the MD could exert differential effects on cognition depending on gender. Studies with both male and female participants show significant results; however, these results are attenuated among female only participants. Similar findings are shown in animal studies, researchers tend to use male animals more than females given that females are more diverse due to hormonal changes and are less likely to show significant results [70].

Another possible reason for this inconsistency in results in the non-Mediterranean region could be that the MD adherence scores were developed to assess dietary intake of people living in the Mediterranean area. Better results would be displayed if the scores were tailored to different populations taking into account the food intake and availability in that particular country. Another reason could be that the Mediterranean diet is observed as a lifestyle rather than just a diet. This lifestyle includes people eating together, and food is considered a social event that brings family and friends together. People in the Mediterranean region are also physically active, and conduct moderate physical activity for at least 30 min per day [71]. Physical activity also exerts a protective role against cognitive decline [72]. This shows that studies examining the effect of diet on cognition need to take physical activity as a possible confounder to this association, and physical activity data needs to be collected.

Collectively, 10 cohort studies, including 35,618 participants included in this review show that adherence to the MD could have a preventative effect on cognitive decline over time. All of these studies had baseline data available and used numerous cognitive tests that assessed the following cognitive domains: concentration, orientation, attention, calculation, recall, language, episodic memory, verbal memory, working memory, long-term memory, visual memory, abstract thinking, category fluency, semantic verbal fluency, tracking and motor speed. Overall, cohort studies show promising effects in which the MD could be an easy and sustainable method in delaying and in some cases preventing dementia. Both RCTs included in this review showed significant results; however, they examined the effect of a MD supplemented with EVOO or nuts. Therefore, the protective association could be due to dietary confounders such as olive oil or nuts rather than the MD. Nuts and olive oil have a high content of anti-oxidants and anti-inflammatory factors which aid in reducing the risk of cognitive decline [49,50]. Both RCTs only included participants at high risk of CVD. Control groups in both RCT were following a low fat diet, maybe the inclusion of a subgroup that consumed the MD as is without any additional intake of olive oil or nuts would have better displayed the effect of the traditional MD on cognition. Nonetheless, these RCTs, given the study design they used and the long period of follow up, provide a base for future randomized trials show the efficacy of the MD on dementia.

## 5. Strengths and Limitations

Our review is unique to the published literature as it investigated the potential differential effect of the MD on cognition in Mediterranean versus non-Mediterranean countries, based on geographical region and ethnicity of study participants. This is important when considering promotion of the adoption of the MD in non-Mediterranean countries to prevent cognitive decline, where adherence rates may differ. This may occur due to different levels of access to MD components or other factors that have not yet been investigated. Our review also considered the MD pattern holistically, rather than the association between specific macronutrient or micronutrient intakes and cognitive function. Other reviews utilize a different approach. Petersson et al. (2016) [73] included 31 articles that linked MD to cognition and AD. Results were reported by study design and did not include study region [73]. Panza et al. (2004) did not explore the intake of the Mediterranean dietary pattern as a whole, but examined intakes of macronutrients (fat and protein) and MUFA and their effect on cognitive function [74]. Several other reviews and meta-analyses have investigated the effect of the MD on AD specifically [75,76]. All of these reviews concluded that the MD was associated with lower rates of cognitive decline, and lower risk of mild cognitive impairment, dementia and AD [76,77,78,79,80,81]. Results from a further 17 articles show that the MD may improve brain health and cognition by decreasing the risk of cardiovascular diseases [82]. Evidence is also reinforced by three meta-analyses that showed an association between the Mediterranean diet and cognitive function [77,83,84].

What differentiates this review is that it was not limited to a specific cognitive domain and included studies that used various cognitive tools. The use of broad inclusion criteria aided in developing a more comprehensive and complete idea about the effect of the MD on cognitive decline. Databases were searched twice to ensure that all published articles in the literature have been examined for eligibility. The quality assessment method used in this review was developed by the Academy of Nutrition and Dietetics and it is a detailed and accurate tool to assess the validity and relevance of articles read. Another strength is that we only included articles that scored neutral or positive on the quality assessment criteria and articles that scored a negative were excluded from this review. Reporting of results was completed in accordance with the Preferred Reporting Items for Systematic Reviews and Meta-Analyses (PRISMA) guidelines; these guidelines are considered an ideal evidence-based method to report systematic reviews.

This review had some limitations that might have affected the reporting of our results. Dietary assessment methods varied from one study to the other, and this might have an effect on the outcomes and conclusions of the studies. For example, some studies used 24 h recalls others used food frequency questionnaires to assess long term dietary intake of the participants (Table 2). The 24 h-recall relies on a trained interviewer’s skills and ability to facilitate portion sizes as well as helping the participant in remembering what they consumed. However, this method is only considered accurate if it is done on various days to better represent usual intake and overcome day to day dietary variations. This method is subject to interviewer bias and reporting bias, nonetheless, an updated standardized version of the 24 h-recall, the multiple pass, aid in overcoming these biases by standardizing data collection [85]. The FFQ can be completed by a trained interviewer or be self-administered. This method is also subject to interviewer bias and reporting bias; however, better results are guaranteed when conducted by a trained interviewer [85]. Another variation is that some studies included in this review conducted these dietary assessments via a face-to-face interview whereas others conducted the assessments via a phone call, yet both methods have been show to have small discrepancies among one another and are considered accurate [86]. Moreover, different scores are used to calculate adherence to the Mediterranean diet, Trichopoulou’s 0–9 MD score and Panagiotakos’s 0–55 MD score. As indicated in this review, both scores have been extensively used in the literature and are shown to be a reliable method of assessing adherence to the traditional MD [17,65]. Results from this review show that the MD scoring criteria used did not affect the association between MD and dementia. Another confounding factor could be the cognitive test used; for example, in the 31 articles included in this review, around 20 different cognitive tests were conducted to assess cognitive function or incidence of dementia.

## 6. Conclusions

Dementia not only affects individuals and their families, but it also exerts immense social and economical impacts. The total cost of dementia in 2015 was 818 billion USD [87]. Most of the studies that examined the effectiveness of the MD on non-communicable diseases, specifically in the older adult population, showed significant results. MD was related to lower risk of CVD, metabolic syndrome, mortality and better mental health. This effect is mainly due to the Mediterranean diet’s high content of B vitamins, folic acid and omega 3 fatty acids; all of which contribute an anti-inflammatory and anti-oxidative function [88]. The MD has been shown to be one of the most nutritionally adequate diets, and people with higher MD adherence scores have a better nutrient profile. Countries in Europe, residing near the Mediterranean Sea, have better nutrient intake qualities, specifically related to: zinc, iron, vitamins B12 and D, folic acid, calcium, selenium and iodine [89]. Most of the aforementioned single nutrients may play a role in improving cognitive function and lowering the risk of AD; however, more studies are needed to have conclusive results [90,91,92,93,94,95,96,97].

Given that there is no direct treatment for dementia, it is fundamental to invest in finding a way to delay its prognosis and reduce the risk of developing it. The Mediterranean diet has been shown to aid in reducing this risk in a cost effective and sustainable manner [98]. The Mediterranean diet lowers the risk of cognitive decline by reducing the risk of developing CVDs, a known risk factor for dementia, and it also has an anti-inflammatory and anti-oxidative effect [99]. The primary aim of this review was to summarize and analyze the literature based research examining the effect of the MD on cognitive function. Our results show that MD does not only play a protective role against cognitive decline, but it might also decrease the risk of developing AD. Various observational studies have shown the beneficial effect of the MD against dementia; however, more randomized controlled trials could aid in concluding long-term effects and strengthen the existing literature. Given the heterogeneity of cognitive tests and dietary outcome measures used, it is important to have a consensus on the diagnosis of cognitive function and the assessment of diet in order to overcome some discrepancies in the literature. Overcoming these gaps will aid in developing a promising, well-structured and comprehensive intervention that includes the MD as a beneficial tool against cognitive decline.

## Figures and Tables

**Table 1:** Summary of the study designs of all articles included in this systematic review.

Authors/Year/Country	Study Quality Score	Study Design	Participants (*n*; Age Mean, Mean ± SD, or Range)	Control Group	Intervention	Follow Up Period	Dietary Measure	Cognitive Outcome Measure (s)
**Studies linking MD to cognitive function**								
Chan et al., 2013 [34] China	0	Cross-sectional	3670 men: 72 ± 4.8 years women: 71.6 ± 4.8 years				0–9 MD score	CSI-D
Crichton et al., 2013 [35] Australia	0	Cross-sectional	1183 50.6 ± 5.8 years				0–9 MD score	CFQ and MFQ
Corley et al., 2013 [36] Scotland	0	Cross-sectional	882 69.5 ± 0.8 years				0–9 MD score	MMSE NART WTAR
Ye et al., 2013 [37] USA	+	Cross-sectional	1269 57.3 ± 7.6 years				0–9 MD score	MMSE
Katsiardanis et al., 2013 [38] Greece	+	Cross-sectional	557 65+ years				0–55 MD score	MMSE
Zbeida et al., 2014 [39] USA	0	Cross-sectional	4577 NHANES 71.19 ± 7.78 years MABAT ZAHAV 74.9 ± 6.25 years				0–9 MD score	Wechsler adult intelligence scale
Martínez-Lapiscina et al., 2013 [40] Spain	+	Randomized controlled trial	522, high CVD risk age 74.6 ± 5.7 years	advised to reduce all types of fat	MD+ EVOO (1 L/week) or MD + 30 g/day of raw, unprocessed mixed nuts	6.5 years		MMSE CDT
Valls-Pedret et al., 2015 [41] Spain	+	Randomized controlled trial	447 high CVD risk mean 66.8 years	advised to reduce dietary fat	MD + EVOO (1 L/week), or MD + mixed nuts (30 g/day)	4.1 years Range (1.0–8.8)		MMSE RAVLT, Animals Semantic Fluency, Wechsler Adult Intelligence Scale, Wechsler Memory Scale, CTT
Cherbuin et al., 2012 [42] Australia	+	Cohort	1528 62.54 ± 1.52 years			4 years	0–9 MD score	International Consensus Criteria, CDR
Haring et al., 2016 [43] USA	+	Cohort	6425 women 65–79 years			9.11 years	0–9 MD score	Consortium to Establish a Registry for Alzheimer’s Disease battery of neuropsychologic tests 3MS
Samieri et al., 2013 [44] USA	+	Cohort	16,058 women 74.3 ± 2.3 years			13 years	0–9 MD score	TICS EBMT Delayed recall of the TICS 10-word list category fluency; digit span-backward
Samieri et al., 2013 [45] USA	0	Cohort	6174 women 72 ± 4.1 years			5 years	0–9 MD score	TICS EBMT; Delayed recall of the TICS ten-word list Category fluency
Vercambre et al., 2012 [46] USA	0	Cohort	2504 women with prevalent vascular disease or more than 3 coronary risk factors 71.9 ± 3.9 years low MD score 72.5 ± 4.3 years middle MD score 72.6 ± 4.0 years High MD score			5.4 years (range 4.1–6.1)	0–9 and 0–55 MD score	TICS 10-word list East Boston Memory Category fluency
Gardener et al., 2015 [47] Australia	0	Cohort	527 69.3 ± 6.4 years			3 years	0–9 MD score	Battery assessed six cognitive domains (verbal memory, visual memory, executive function, language, attention and visuospatial functioning
Psaltopoulou et al., 2008 [48] Greece	0	Cohort	743 >65 years			Median 8 years, range (6–13)	0–9 MD score	MMSE
Qin et al., 2015 [49] China	+	Cohort	1650 55+ years			5.3 years	0–9 MD score	Immediate and delayed recall of a 10-word list; counting backward and serial 7’s
Feart et al., 2009 [50] France	0	Cohort	1410 75.9 (range, 67.7–94.9) years			4.1 years	0–9 MD score	BVRT FCSRT MMSE IST
Kesse-Guyot et al., 2013 [51] France	0	Cohort	3083 52.0 ± 4.6 years			13 years	0–9 MD score 0–100 MSDPS	RI- 48 (Rappel indice’ (cued recall)-48 items)
Galbete et al., 2015 [52] Spain	0	Cohort	823 62 ± 6 years			8 years	0–9 MD score	TICS-m
Koyama et al., 2015 [53] USA	+	Cohort	2326 74.6 ± 2.9 years			7.9 ± 0.1 years	0–55 MD score	3MS
Tangney et al., 2011 [54] USA	0	Cohort	3790 75.4 ± 6.2 years			7.6 years	0–55 MD score	East Boston tests of immediate and delayed recall MMSE Symbol Digit Modalities Test
Tangney et al., 2014 [55] USA	0	Cohort	826 81.5 ± 7.1 years			4.1 years	0–55 MD score	19 cognitive tests
Trichopoulou et al., 2015 [56] Greece	0	Cohort	401 mean = 74 years			6.6 years	0–9 MD score	MMSE
Wengreen et al., 2013 [57] USA	+	Cohort	3831 73.8; 10.2 74.1; 10.2 74.4; 10.0 74.0; 9.7 74.2; 9.7 per 5 MD quintiles			10.6 years	0–9 MD score	3MS
Tsivgoulis et al., 2013 [58] USA	+	Cohort	17,478 64.4 ± 9.1 years			4.0 ± 1.5 years	0–9 MD score	SIS
**Studies linking MD to AD development and mortality**								
Gardener et al., 2012 [59] Australia	0	Cross-sectional	970 71.72 ± 7.86 years				0–9 MD score	MMSE Logical Memory II California Verbal Learning Test II Delis-Kaplan Executive Function System Verbal Fluency
Scarmeas et al., 2009 [60] USA	+	Cohort	1393 76.9 ± 6.5 years			4.3 ± 2.7 years	0–9 MD score	Alzheimer’s incidence rate CDR
Olsson et al., 2015 [61] Sweden	+	Cohort	1038 men 71 ± 0.6 years			12 years	0–8 MD score	Alzheimer’s incidence rate NINCDS-ADRDA DSM-IV criteria MMSE
Scarmeas et al., 2007 [62] USA	+	Cohort	192 82.9 ± 7.7 years			4.4 ± 3.6 years	0-9 MD score	Mortality rate
Morris et al., 2015 [63] USA	0	Cohort	923 58–98 years			Average 4.5 years	0–55 MD score	Alzheimer’s incidence rate
Gu et al., 2010 [64] USA	0	Cohort	1219 76.7 ± 6.4 years			3.8 ± 1.3 years	0–9 MD score	DSM III R NINCDS-ADRDA

Abbreviations list for Table 1; CSI-D: Community Screening Instrument for Dementia; CFQ: Cognitive Failures Questionnaire; MFQ: Memory Functioning Questionnaire; MMSE: Mini-Mental State Examination; NART: National Adult Reading Test; WTAR: Wechsler Test of Adult Reading; CDT: Clock Drawing Test; RAVLT: Rey Auditory Verbal Learning Test; CTT: Color Trail Test; CDR: Clinical Dementia Rating scale; 3MS: Modified Mini-Mental State Examination; TICS: Telephone Interview of Cognitive Status; EBMTL: East Boston Memory test; BVRT: Benton Visual Retention Test; FCSRT: Free and Cued Selective Reminding Test; IST: Isaacs Set Test; RI- 48 (Rappel indices’ (cued recall)-48 items): 48 items Free and Cued Recall; DSM III R: Diagnostic and Statistical Manual of Mental; NINCDS-ADRDA: National Institute of Neurological and Communicative Disorders and Stroke and the Alzheimer’s Disease and Related Disorders Association.

**Table 2:** Summary of the results and limitations of all articles included in this systematic review.

Study	Results	Limitations
Chan et al., 2013 [34]	MD was not associated with cognitive function	Cross-sectional study (cannot establish causality); cognitive status was self-reported; participants were highly educated
Crichton et al., 2013 [35]	MD was not associated with cognitive function	Cross-sectional study (cannot establish causality; cognitive status was self-reported; inclusion exclusion criteria not clearly stated
Corley et al., 2013 [36]	MD was associated with improved cognitive function; however, the association was no longer significant after adjusting for childhood IQ and socio-economic status	Self-selecting sample; inclusion exclusion criteria not clearly stated; response rate not stated
Ye et al., 2013 [37]	MD was associated with improved MMSE scores (OR of cognitive impairment = 0.87, 95% CI (0.80–0.94)	Cross-sectional study (cannot establish causality)
Katsiardanis et al., 2013 [38]	MD was associated with improved MMSE scores in male; OR of cognitive impairment = 0.88, 95% CI (0.80–0.98); however, no significant association was observed among females	Cross-sectional study (cannot establish causality)
Zbeida et al., 2014 [39]	MD was associated with improved cognitive function in both NHANES and MABAT-ZAHAV cohorts, *p* < 0.001 and 0.008 respectively	Cross-sectional study (cannot establish causality); used a 24 h recall which does not represent usual intake; inclusion exclusion criteria not clearly stated; sources of funding not mentioned
Martínez-Lapiscina et al., 2013 [40]	MD + EVOO and MD + Nuts diet were significantly associated with better cognitive function. MD + EVOO group’s mean global cognitive function scores differences from the control group (+0.62, 95% CI (0.18–1.05), *p* = 0.005 for MMSE, and +0.51, 95% CI (0.20–0.82), *p* = 0.001 for CDT). MD + Nuts group’s differences from the control group +0.57, 95% CI (0.11–1.03), *p* = 0.015 for MMSE and +0.33, 95% CI (0.003–0.67), *p* = 0.048 for CDT)	Participants had CVD risk factors which may improve effect size seen; intervention was MD plus EVOO or nuts; no baseline data
Valls-Pedret et al., 2015 [41]	MD + EVOO was significantly associated with RAVLT and color trail test, *p* value = 0.04 and 0.045 respectively. MD + EVOO was not associated with other cognitive tests measured MD + Nuts was significantly associated with better composite memory, and MD + EVOO was was significantly associated with better global cognition	Participants had CVD risk factors; intervention was MD plus EVOO or nuts; funding may have caused a conflict of interest
Cherbuin et al., 2012 [42]	MD was not associated with cognitive function	No limitations identified
Haring et al., 2016 [43]	MD was not associated with cognitive function	Female participants only; baseline dietary data only
Samieri et al., 2013 [44]	MD was associated with cognitive function. Cross-sectional analysis showed that higher MD was associated with better TICS scores, global cognition and verbal memory, *p* = 0.004, 0.002 and <0.001 respectively. No effect of MD on cognitive function over time	Female only; highly educated; telephone assessment; unclear reliability
Samieri et al., 2013 [45]	MD was not associated with cognitive function	Female only; highly educated; telephone assessment
Vercambre et al., 2012 [46]	MD was not associated with cognitive function	Female participants only; participants had CVD risk factors; cognitive function assessed via telephone; baseline dietary data only
Gardener et al., 2015 [47]	MD was associated with executive cognitive function only among APOE allele carrier; change in cognitive function = 8.6%, *p* < 0.01	Cohort medians for food intakes were used instead of traditional medians
Psaltopoulou et al., 2008 [48]	MD was not associated with cognitive function	No baseline data; exclusion criteria not clearly stated
Qin et al., 2015 [49]	MD was associated with slower cognitive decline, β = 0.042, 95% CI (0.002–0.081)	24 h recall which does not represent usual intake
Feart et al., 2009 [50]	MD was associated with better cognition, MMSE errors, β = −0.006, 95% CI (−0.01, −0.0003) per 1-unit increase in MD score MD was not associated with other cognitive tests (IST, BVRT, or FCSRT) and the risk of developing dementia	Selection bias, participants with missing data were significantly different than those with available data
Galbete et al., 2015 [51]	MD was associated with better cognition, higher MD scores had lower rates of cognitive decline, *p* = 0.011	Participants were highly educated and did not represent general public
Kesse-Guyot et al., 2013 [52]	MD was associated with lower phonemic fluency score, *p* = 0.048 and a lower backward digit span score, *p* = 0.03	No baseline data; low response rate
Koyama et al., 2015 [53]	MD was associated with better cognition among African-American participants, high MD was associated with better 3MS scores, difference = 0.22, 95% CI (0.05–0.39), *p* = 0.01	African-Americans are at a higher risk of CVD
Tangney et al., 2011 [54]	MD was associated with better cognition, MD was associated with slower rates of cognitive decline β = 0.0014 per 1-point increase, *p* = 0.0004	24 h recall which does not represent usual intake
Tangney et al., 2014 [55]	MD was associated with better cognition. In linear analysis, MD was associated with better Global cognition, Episodic memory and Semantic memory, β = 0.002, 0.003, 0.003 and *p* = 0.01, 0.02 and 0.02 respectively In the categorical analysis, MD was associated with better working memory, β = 0.033, *p* = 0.01	Sample does not represent the general public
Trichopoulou et al., 2015 [56]	MD was associated with better cognition. Low MMSE scores were associated with low adherence to the MD; 20% of participants with low MMSE adhered well to the MD (MD score 6–9), as compared to a 41% in the high MMSE group. OR comparing high to low MD adherence was 0.46, 95 % CI (0.25–0.87) and 0.34, 95 % CI (0.13–0.89) for mild versus no decline and substantial versus no decline respectively. For highest MD scores OR = 0.46, 95 % CI (0.25–0.87) and 0.34, 95 % CI (0.13–0.89) for mild and substantial cognitive decline respectively	High rates of withdrawals
Wengreen et al., 2013 [57]	MD was associated with better cognition. Participants with the highest MD scores scored 1.4 times higher on the 3MS cognitive score, *p* = 0.0014	No limitations identified
Tsivgoulis et al., 2013 [58]	MD was associated with better cognition only among non-diabetics. Higher MD scores were associated with lower risk of incident cognitive impairment, OR = 0.81, 95% CI (0.70–0.94)	Dietary intake was only assessed at baseline
Gardener et al., 2012 [59]	MD was associated with better cognition. Participants with AD and mild cognitive impairment had lower MD scores, *p* < 0.001 and <0.05 respectively. Each 1-unit increase was associated with 13–19% and 19–36% lower risk of being in the MCI and AD group respectively. MD was also linearly correlated with MMSE scores, *p* = 0.014.	Some under-reporting in FFQ; did not collect participant’s country of origin; inclusion exclusion criteria not clearly stated
Scarmeas et al., 2009 [60]	MD was associated with lower risk of developing AD. Participants within the middle and highest tertile of MD had a 45%, 95% CI (0.34–0.90) and 48%, 95% CI (0.53–0.95) of developing AD.	No limitations detected
Olsson et al., 2015 [61]	MD was not associated with cognitive function	Single 3-day food records with no follow-up data; male only
Scarmeas et al., 2007 [62]	MD was associated with lower mortality rates among AD patients; participants with highest adherence to MD had a mortality HR = 0.27, 95% CI (0.10–0.69)	Urban setting
Morris et al., 2015 [63]	MD was associated with lower risk of developing AD. Participants in the highest MD tertile had a HR of developing AD = 0.46, 95% CI (0.29, 0.74)	Participants were volunteers who are usually more health aware
Gu et al., 2010 [64]	MD was associated with lower risk of developing AD. Participants in the highest MD tertile had a 34% less risk of developing AD, *p* = 0.04	Characteristics of participants that loss follow-up were different than those who remained; inclusion criteria was not clear

**Table 3:** Follow up periods of all cohorts included in this review.

Cohort	Follow Up Time (Years)	Cohort	Follow Up Time (Years)
**Studies linking MD to cognitive function**			
**Studies that detected significance**		**Studies that didn’t detected significance**	
Tsivgoulis et al., 2013 [48]	4	Gardener et al., 2015 [47]	3
Tangney et al., 2014 [55]	4.1	Cherbuin et al., 2012 [42]	4
Feart et al., 2009 [50]	4.1	Samieri et al., 2013 [45]	5
Qin et al., 2015 [49]	5.3	Vercambre et al., 2012 [46]	5.4
Trichopoulou et al., 2015 [56]	6.6	Psaltopoulou et al., 2008 [48]	8
Tangney et al., 2011 [54]	7.6	Haring et al., 2016 [43]	9.1
Koyama et al., 2015 [53]	7.9	Samieri et al., 2013 [44]	13
Galbete et al., 2015 [52]	8		
Wengreen et al., 2013 [57]	10.6		
Kesse-Guyot et al., 2013 [51]	13		
**Studies linking MD to AD development and mortality**			
**Studies that detected significance**		**Studies that didn’t detected significance**	
Gu et al., 2010 [64]	3.8	Olsson et al., 2015 [61]	12
Scarmeas et al., 2009 [60]	4.3		
Scarmeas et al., 2007 [62]	4.4		
Morris et al., 2015 [63]	4.5		

## Supplementary Materials

The supplementary file is available online at www.mdpi.com/2072-6643/9/7/674/s1.
