# Supplementary material for: The Association between the Mediterranean Dietary Pattern and Cognitive Health: A Systematic Review
Source: Nutrients. 2017 Jun 28;9(7):674. doi: 10.3390/nu9070674 (PMC5537789; doi:10.3390/nu9070674)
Supplement: Supplementary file 1 [file nutrients-09-00674-s001.pdf]

Supplementary material 1

Adapted from the Academy of Nutrition and Dietetics Evidence Analysis Library Quality Criteria Checklist for Primary Research

Appendix 8: Quality Criteria Checklist: Primary Research

Symbols Used

\+ **Positive:** Indicates that the report has clearly addressed issues of inclusion/exclusion, bias, generalizability, and data collection and analysis.

-- **Negative:** Indicates that these issues have not been adequately addressed.

∅ **Neutral:** Indicates that the report is neither exceptionally strong nor exceptionally weak.

***Quality Criteria Checklist*: Primary Research**

RELEVANCE QUESTIONS	
1. Would implementing the studied intervention or procedure (if found successful) result in improved outcomes for the patients/clients/population group? (NA for some Epi studies)	Yes No Unclear N/A
2. Did the authors study an outcome (dependent variable) or topic that the patients/clients/population group would care about?	Yes No Unclear N/A
3. Is the focus of the intervention or procedure (independent variable) or topic of study a common issue of concern to dietetics practice?	Yes No Unclear N/A
4. Is the intervention or procedure feasible? (NA for some epidemiological studies)	Yes No Unclear N/A
If the answers to all of the above relevance questions are "Yes," the report is eligible for designation with a plus (+) on the Evidence Quality Worksheet, depending on answers to the following validity questions.	
VALIDITY QUESTIONS	
**1. Was the <u>research question</u> clearly stated?**<br>1.1 Was the specific intervention(s) or procedure (independent variable(s)) identified?<br>1.2 Was the outcome(s) (dependent variable(s)) clearly indicated?<br>1.3 Were the target population and setting specified?	Yes No Unclear N/A
**2. Was the <u>selection</u> of study subjects/patients free from bias?**<br>2.1 Were inclusion/exclusion criteria specified (e.g., risk, point in disease progression, diagnostic or prognosis criteria), and with sufficient detail and without omitting criteria critical to the study?<br>2.2 Were criteria applied equally to all study groups?<br>2.3 Were health, demographics, and other characteristics of subjects described?<br>2.4 Were the subjects/patients a representative sample of the relevant population?	Yes No Unclear N/A
**3. Were <u>study groups comparable</u>?**<br>3.1 Was the method of assigning subjects/patients to groups described and unbiased? (Method of randomization identified if RCT)<br>3.2 Were distribution of disease status, prognostic factors, and other factors (e.g., demographics) similar across study groups at baseline?<br>3.3 Were concurrent controls used? (Concurrent preferred over historical controls.)<br>3.4 If cohort study or cross-sectional study, were groups comparable on important confounding factors and/or were preexisting differences accounted for by using appropriate adjustments in statistical analysis?<br>3.5 If case control study, were potential confounding factors comparable for cases and controls? (If case series or trial with subjects serving as own control, this criterion is not applicable. Criterion may not be applicable in some cross-sectional studies.)<br>3.6 If diagnostic test, was there an independent blind comparison with an appropriate reference standard (e.g., "gold standard")?	Yes No Unclear N/A
**4. Was method of handling <u>withdrawals</u> described?**<br>4.1 Were follow up methods described and the same for all groups?<br>4.2 Was the number, characteristics of withdrawals (i.e., dropouts, lost to follow up, attrition rate) and/or response rate (cross-sectional studies) described for each group? (Follow up goal for a strong study is 80%.)<br>4.3 Were all enrolled subjects/patients (in the original sample) accounted for?<br>4.4 Were reasons for withdrawals similar across groups?	Yes No Unclear N/A

4.5	If diagnostic test, was decision to perform reference test not dependent on results of test under study?	
**5.**	**Was <u>blinding</u> used to prevent introduction of bias?**	Yes No Unclear N/A
5.1	In intervention study, were subjects, clinicians/practitioners, and investigators blinded to treatment group, as appropriate?	
5.2	Were data collectors blinded for outcomes assessment? (If outcome is measured using an objective test, such as a lab value, this criterion is assumed to be met.)	
5.3	In cohort study or cross-sectional study, were measurements of outcomes and risk factors blinded?	
5.4	In case control study, was case definition explicit and case ascertainment not influenced by exposure status?	
5.5	In diagnostic study, were test results blinded to patient history and other test results?	
**6.**	**Were <u>intervention</u>/therapeutic regimens/exposure factor or procedure and any comparison(s) described in detail? Were <u>intervening factors</u> described?**	Yes No Unclear N/A
6.1	In RCT or other intervention trial, were protocols described for all regimens studied?	
6.2	n observational study, were interventions, study settings, and clinicians/provider described?	
6.3	Was the intensity and duration of the intervention or exposure factor sufficient to produce a meaningful effect?	
6.4	Was the amount of exposure and, if relevant, subject/patient compliance measured?	
6.5	Were co-interventions (e.g., ancillary treatments, other therapies) described?	
6.6	Were extra or unplanned treatments described?	
6.7	Was the information for 6.4, 6.5, and 6.6 assessed the same way for all groups?	
6.8	In diagnostic study, were details of test administration and replication sufficient?	
**7.**	**Were <u>outcomes</u> clearly defined and the <u>measurements valid and reliable</u>?**	Yes No Unclear N/A
7.1	Were primary and secondary endpoints described and relevant to the question?	
7.2	Were nutrition measures appropriate to question and outcomes of concern?	
7.3	Was the period of follow-up long enough for important outcome(s) to occur?	
7.4	Were the observations and measurements based on standard, valid, and reliable data collection instruments/tests/procedures?	
7.5	Was the measurement of effect at an appropriate level of precision?	
7.6	Were other factors accounted for (measured) that could affect outcomes?	
7.7	Were the measurements conducted consistently across groups?	
**8.**	**Was the <u>statistical analysis</u> appropriate for the study design and type of outcome indicators?**	Yes No Unclear N/A
8.1	Were statistical analyses adequately described the results reported appropriately?	
8.2	Were correct statistical tests used and assumptions of test not violated?	
8.3	Were statistics reported with levels of significance and/or confidence intervals?	
8.4	Was "intent to treat" analysis of outcomes done (and as appropriate, was there an analysis of outcomes for those maximally exposed or a dose-response analysis)?	
8.5	Were adequate adjustments made for effects of confounding factors that might have affected the outcomes (e.g., multivariate analyses)?	
8.6	Was clinical significance as well as statistical significance reported?	
8.7	If negative findings, was a power calculation reported to address type 2 error?	
**9.**	**Are <u>conclusions supported by results</u> with biases and limitations taken into consideration?**	Yes No Unclear N/A
9.1	Is there a discussion of findings?	
9.2	Are biases and study limitations identified and discussed?	
**10.**	**Is bias due to study's <u>funding or sponsorship</u> unlikely?**	Yes No Unclear N/A
10.1	Were sources of funding and investigators' affiliations described?	
10.2	Was there no apparent conflict of interest?	

MINUS/NEGATIVE (-)

If most (six or more) of the answers to the above validity questions are "No," the report should be designated with a minus (-) symbol on the Evidence Worksheet.

Table showing the detailed quality assessment for all articles included in this review

Academy of Nutrition and Dietetics Evidence Analysis Library Quality Criteria Checklist for Primary Research

Article	Relevance question				Validity questions										Total
	1	2	3	4	1	2	3	4	5	6	7	8	9	10	
**Chan et al., 2013**	NA	yes	yes	Yes	yes	no	yes	yes	yes	yes	yes	yes	yes	yes	0
**Cherbuin et al., 2012**	NA	yes	yes	Yes	yes	yes	yes	yes	yes	yes	yes	yes	yes	yes	+
**Gardener et al., 2012**	NA	yes	yes	Yes	yes	no	yes	yes	yes	yes	yes	yes	yes	yes	0
**Ye et al., 2013**	NA	yes	yes	Yes	yes	yes	yes	NA	yes	yes	yes	yes	yes	yes	+
**Katsiardanis et al., 2013**	NA	yes	yes	Yes	yes	yes	yes	yes	yes	yes	yes	yes	yes	yes	+
**Zbeida et al., 2014**	NA	yes	yes	Yes		Unclear	yes	yes	yes	yes	yes	yes	yes	Unclear	0
**Martínez-Lapiscina et al., 2013**	yes	yes	yes	Yes	yes	yes	yes	yes	yes	yes	yes	yes	yes	yes	+
**Valls-Pedret et al., 2015**	yes	yes	yes	Yes	yes	yes	yes	yes	yes	yes	yes	yes	yes	no	+
**Crichton et al., 2013**	NA	yes	yes	Yes	yes	no	yes	yes	yes	yes	yes	yes	yes	yes	0
**Haring et al., 2016**	NA	yes	yes	Yes	yes	yes	yes	yes	yes	yes	yes	yes	yes	yes	+
**Qin et al., 2015**	NA	yes	yes	Yes	yes	yes	yes	yes	yes	yes	yes	yes	yes	yes	+
**Scarmeas et al., 2009**	NA	yes	yes	Yes	yes	yes	yes	yes	yes	yes	yes	yes	yes	yes	+
**Feart et al., 2009**	NA	yes	yes	Yes	yes	Unclear	yes	yes	yes	yes	yes	yes	yes	yes	0
**Galbete et al., 2015**	NA	yes	yes	Yes	yes	Unclear	yes	yes	yes	yes	yes	yes	yes	yes	0

**Gu et al., 2010**	NA	yes	yes	Yes	yes	Unclear	yes	yes	yes	yes	yes	yes	yes	yes	0
**Kesse-Guyot et al., 2013**	NA	yes	yes	Yes	yes	yes	yes	no	yes	yes	yes	yes	yes	yes	0
**Koyama et al., 2015**	NA	yes	yes	Yes	yes	yes	yes	no	yes	yes	yes	yes	yes	yes	+
**Morris et al., 2015**	NA	yes	yes	Yes	yes	no	yes	NA	yes	yes	yes	yes	yes	yes	0
**Samieri et al., 2013 (MD)**	NA	yes	yes	Yes	yes	no	yes	yes	yes	yes	yes	yes	yes	yes	0
**Samieri et al., 2013 (longterm)**	NA	yes	yes	Yes	yes	no	yes	NA	yes	yes	yes	yes	yes	yes	0
**Tangney et al., 2011**	NA	yes	yes	Yes	yes	no	yes	yes	yes	yes	yes	yes	yes	yes	0
**Tangney et al., 2014**	NA	yes	yes	Yes	yes	no	yes	no	yes	yes	yes	yes	yes	yes	0
**Trichopoulou et al., 2015**	NA	yes	yes	Yes	yes	no	yes	NA	yes	yes	yes	yes	yes	yes	0
**Vercambre et al., 2012**	NA	yes	yes	Yes	yes	no	yes	NA	yes	no	yes	yes	yes	yes	0
**Wengreen et al., 2013**	NA	yes	yes	Yes	yes	yes	yes	NA	yes	yes	yes	yes	yes	yes	+
**Corley et al., 2013**	NA	yes	yes	Yes	yes	no	yes	no	yes	yes	yes	yes	yes	yes	0
**Tsivgoulis et al., 2013**	NA	yes	yes	Yes	yes	yes	yes	yes	yes	yes	yes	yes	yes	yes	+
**Gardener et al., 2015**	NA	yes	yes	Yes	yes	yes	yes	yes	yes	yes	no	yes	yes	yes	0
**Olsson et al., 2015**	NA	yes	yes	Yes	yes	yes	yes	yes	yes	yes	no	yes	yes	yes	0
**Scarmeas et al., 2007**	NA	yes	yes	Yes	yes	yes	yes	yes	yes	yes	yes	yes	yes	yes	+
**Psaltopoulou et al, 2008**	NA	yes	yes	Yes	yes	Unclear	yes	yes	yes	yes	yes	yes	yes	yes	0
